# Design and Realization of a High-Q Grounded Tunable Active Inductor for 5G NR (FR1) Transceiver Front-End Applications

**DOI:** 10.3390/s25103070

**Published:** 2025-05-13

**Authors:** Sehmi Saad, Aymen Ben Hammadi, Fayrouz Haddad

**Affiliations:** 1 Aix Marseille Université, Technopôle de Château Gombert, IM2NP UMR 7334, 13397 Marseille, France; 2 Dynamics RF/mmW Consulting and Design, 39 Avenue du Vercors, 38600 Grenoble, France

**Keywords:** grounded active inductor, wide tuning range, radio frequency, CMOS

## Abstract

This paper presents a wide-tuning-range, low-power tunable active inductor (AI) designed and fabricated using 130 nm CMOS technology with six metal layers. To achieve high performance with a relatively small silicon area and low power consumption, the AI structure is carefully designed and optimized using a cascode stage, a feedback resistor, and multi-gate finger transistors. In the proposed circuit topology, inductance tuning is realized by adjusting both the bias current and the feedback resistor. The performance of the circuit is evaluated in terms of tuning range, quality factor, power consumption, and chip area. The functionality of the fabricated device is experimentally validated, and the fundamental characteristics of the active inductor are measured over a wide frequency range using a Cascade GSG probe, with results compared to simulations. Experimental measurements show that, under a 1 V supply, the AI achieves a self-resonant frequency (SRF) of 3.961 GHz and a quality factor (Q) exceeding 1586 at 2.383 GHz. The inductance is tunable between 6.7 nH and 84.4 nH, with a total power consumption of approximately 2 mW. The total active area, including pads, is 345 × 400 µm^2^.

## 1. Introduction

The rapid evolution of 5G New Radio (NR) has significantly transformed wireless communication by enabling higher data rates, lower latency, and improved spectral efficiency. A key component of this advancement is Frequency Range 1 (FR1), spanning from 410 MHz to 7125 MHz, which serves as the backbone for early 5G deployment [1,2,3]. The FR1 spectrum offers notable advantages due to its favorable propagation characteristics, compatibility with existing cellular infrastructure, and support for multiple duplexing schemes, including Frequency Division Duplex (FDD) and Time Division Duplex (TDD) [4]. As wider bandwidths and higher operating frequencies become increasingly important, the design of high-performance RF front-end circuits faces new challenges, necessitating the development of compact, tunable, and power-efficient components.

Inductive elements are fundamental building blocks in RF front-end circuits, playing critical roles in applications such as low-noise amplifiers (LNAs), voltage-controlled oscillators (VCOs), impedance matching networks, and tunable filters. While passive spiral inductors have traditionally been favored for their simplicity, they present several drawbacks, including large silicon area requirements, limited tunability, and significant Q-factor degradation due to substrate losses [5]. These limitations become more pronounced at higher frequencies, making passive inductors less suitable for 5G NR (FR1) applications. To address these issues, recent research has increasingly focused on inductor-less architectures [6]. In this context, active inductors have emerged as promising alternatives, offering wide tunability, high inductance density, and improved integration with standard CMOS processes. However, practical deployment of active inductors is hindered by challenges such as low Q-factors, increased noise, and higher power consumption.

In the literature, a wide range of active inductance simulators has been proposed, classified based on their active/passive component configurations and whether they generate grounded or floating inductances. Examples include:

− Current-feedback operational amplifiers (CFOAs) [7,8,9];
− Current differencing transconductance amplifiers (CDTAs) [10];
− Voltage differencing differential input-buffered amplifiers (VD-DIBAs) [11,12];
− Differential voltage current conveyors (DVCCs) [13];
− Differential current conveyors (DCCIIs) [14];
− Fully differential current conveyors (FDCCIIs) [15];
− Second-generation current conveyors (CCIIs) [16];
− Third-generation current conveyors (CCIIIs) [17];
− Differential difference current conveyors (DDCCs) [18,19,20];
− Four-terminal floating nullors (FTFNs) [21];
− Operational transconductance amplifiers (OTAs) [22,23], among others [24].

Among these, OTA-based active inductors, often implemented using gyrator-C topologies, have gained particular prominence due to their low transistor count, simplified architecture, and favorable high-frequency characteristics [23].

Several studies have proposed techniques to enhance the performance of gyrator-C-based active inductors. Negative resistance compensation [25] is one such method, improving the Q-factor by offsetting resistive losses using negative impedance converters (NICs) or cross-coupled transistors, without significant power overhead.

Another widely adopted approach is the cascode topology [26], which improves linearity, frequency response, and circuit stability by stacking transistors to increase output impedance, thus enhancing bandwidth and the Q-factor. Feedback-based techniques, including shunt and series feedback, are also employed to enhance inductance and the Q-factor while maintaining low power dissipation [27,28].

Additionally, advancements in fabrication technologies, such as FinFET [29], silicon-on-insulator (SOI) [30], and gallium nitride (GaN) [31], have reduced substrate losses and parasitic effects, further improving performance. Moreover, tunable biasing allows for dynamic adjustment of inductor characteristics, enabling adaptability to varying frequency and bandwidth requirements in 5G systems.

In this work, an active inductor circuit is proposed, offering a high quality factor and wide tunability, designed to meet the performance and efficiency demands of 5G NR (FR1) applications. Although many CMOS-based active inductor designs have been investigated, relatively few report measured results. This study introduces and experimentally characterizes a grounded CMOS active inductor based on a gyrator-C architecture.

The proposed design is optimized to operate within the sub-6 GHz FR1 spectrum, covering key frequency bands used in 5G. The focus is on addressing the core challenges of active inductor integration in 5G NR systems, with emphasis on achieving a high Q-factor, wide tunability, and low power consumption.

This paper is organized as follows: Section 2 reviews the fundamental principles of grounded gyrator-C active inductors and key design considerations. As well, it presents the proposed circuit topology and outlines the techniques employed to enhance its performance. Section 3 details the measurement results and performance analysis. Section 4 concludes the paper and discusses future research directions.

## 2. Grounded Gyrator-C-Based Tunable Active Inductor

### 2.1. Basic Concept

**Figure 1:**
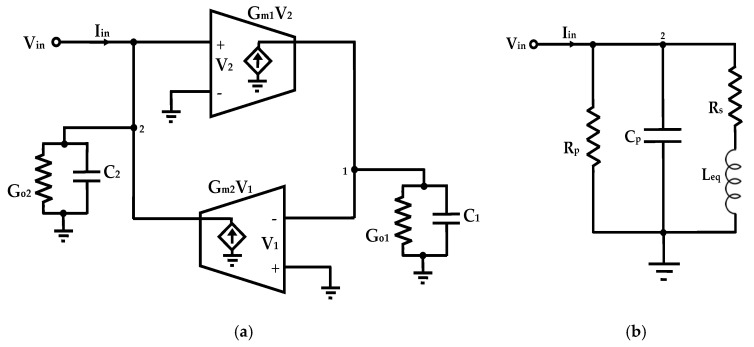
(**a**) Lossy single-ended gyrator-C network-based AIs, and (**b**) the corresponding equivalent RLC model.

A gyrator can be realized by connecting two transconductors in series, where one exhibits positive transconductance and the other negative transconductance. When a capacitor is connected at the output, the resulting configuration forms a gyrator-C network, as illustrated in Figure 1a. By applying Kirchhoff’s Current Law (KCL) at nodes 1 and 2, the input admittance Y_in_ can be derived:

(1) $$Y_{\mathrm{in}}=\frac{I_{\mathrm{in}}}{V_{2}}=s\;C_{2}+G_{o2}+\frac{1}{\frac{s\;C_{1}}{G_{m1}G_{m2}}+\frac{G_{o1}}{G_{m1}G_{m2}}}$$

This expression reveals that the gyrator-C structure emulates the behavior of an equivalent RLC network, as represented in Figure 1b, with the corresponding parameters given by:

(2) $$C_{p}=C_{2},\;R_{p}=\frac{1}{G_{o2}},\;L_{eq}=\frac{C_{1}}{G_{m1}G_{m2}},\;\mathrm{and}\;R_{s}=\frac{G_{o1}}{G_{m1}G_{m2}}$$

where G_o1_ and G_o2_ denote the total input and output conductances of the transconductors at ports 1 and 2, respectively.

From Equation (2), a gyrator-C network can replicate the behavior of an inductor L_eq_ along with its associated parasitic components: parallel resistance R_p_, parallel capacitance C_p_, and series resistance R_s_. The equivalent inductance is directly proportional to the output capacitance C_1_ and inversely proportional to the transconductances G_m1_ and G_m2_, allowing for tunability through adjustment of these parameters. However, the inductive behavior is confined to a specific frequency range, which can be determined by analyzing the input impedance Z_in_, as defined in Equation (3).

(3) $$Z_{\mathrm{in}}=\left(\frac{R_{s}}{L_{\mathrm{eq}}C_{p}}\right)\frac{s\frac{L_{eq}}{R_{s}}+1}{s^{2}+s\left(\frac{1}{R_{p}C_{p}}+\frac{R_{s}}{L_{eq}}\right)+\frac{R_{s}+R_{p}}{R_{p}L_{\mathrm{eq}}C_{p}}}$$

The inductive behavior of a lossy gyrator-C network is limited to a frequency band bounded by the zero frequency ω_z_ and the self-resonant frequency ω_0_. The self-resonant frequency ω_0_ is obtained by ensuring that the phase angle of Z_in_ is zero, which occurs when the phase angles of the numerator and denominator are equal. By setting ∡ Z_in_(jω) = 0 and using s *=* jω, the value of ω_0_ can be derived as:

(4) $$\arctan\;∡\;\;\left(1+s\frac{L_{\mathrm{eq}}}{R_{s}}\right)=\arctan\;∡\;\;\left(\left(\frac{R_{s}+R_{p}}{R_{p}L_{\mathrm{eq}}C_{p}}-\omega^{2}\right)+s\left(\frac{1}{R_{p}C_{p}}+\frac{R_{s}}{L_{\mathrm{eq}}}\right)\right)$$

By solving Equation (4), ω_0_ can be expressed as:

(5) $$\omega_{0}=\sqrt{\frac{1}{\sqrt{L_{\mathrm{eq}}C_{p}}}-{\left(\frac{R_{s}}{L_{\mathrm{eq}}}\right)}^{2}}$$

From Equation (3), the input impedance Z_in_ exhibits a zero at the frequency ω_z_ = R_S_/L_eq_. Additionally, Z_in_ has a pole corresponding to the resonant frequency given by $\omega_{p}=\sqrt{\left(R_{s}+R_{p}\right)/R_{p}L_{\mathrm{eq}}C_{p}}$. Under the condition where R_p_ ≫ R_s_ and R_s_ is considered negligible, the resonant frequency ω_0_ and the pole frequency ω_p_ can be approximated as nearly equal $\omega_{p}=\sqrt{1/L_{\mathrm{eq}}C_{p}}≃\omega_{0}$.

**Figure 2:**
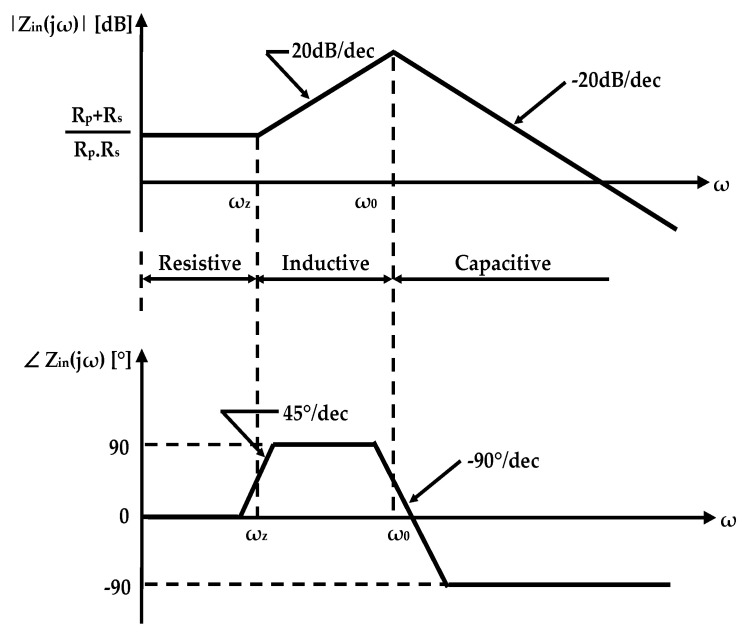
Bode plot of input impedance Z_in_ for a lossy single-ended gyrator-C active inductor.

Figure 2 presents the Bode plots of Z_in_, illustrating the frequency-dependent behavior of the lossy gyrator-C network. The network demonstrates resistive characteristics at frequencies ω < ω_z_, behaves inductively in the range of ω_z_ < ω < ω_0_, and exhibits capacitive behavior for ω > ω_0_ [23].

An additional key parameter of an active inductor is the quality factor Q, which quantifies its efficiency. For linear inductors, including active inductors, Q is defined as the ratio of the imaginary to the real part of the input impedance. Based on the transfer function of Z_in_ in Equation (3), the quality factor for lossy active inductors can be expressed as:

(6) $$Q=\left(\frac{\omega L_{\mathrm{eq}}}{R_{s}}\right)\left(\frac{1-\left(R_{s}^{2}C_{p}/L_{\mathrm{eq}}\right)-\omega^{2}L_{\mathrm{eq}}C_{p}}{1+\left(R_{s}/R_{p}\right)\left(1+{\left(\omega L_{\mathrm{eq}}/R_{s}\right)}^{2}\right)}\right)$$

**Figure 3:**
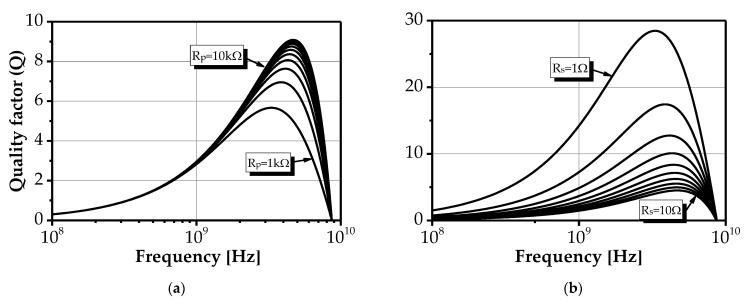
Influence of (**a**) R_p_ and (**b**) R_s_ on the quality factor of AIs, with L_eq_ = 2.4 nH, C_p_ = 140 fF, R_s_ = 5 Ω, and R_p_ = 5 kΩ.

Figure 3 shows the effect of variations in R_p_ and R_s_ on the quality factor of active inductors, using the parameter values L_eq_ = 2.4 nH, C_p_ = 140 fF, R_s_ = 5 Ω, and R_p_ = 5 kΩ. Enhancement in the quality factor is achieved by increasing R_p_ and reducing R_s_.

When the values of series and parallel resistances are comparable, the parallel resistance R_p_ has a more dominant influence on determining the quality factor Q. In advanced deep-submicron technologies, where the transconductance of transistors is significantly greater than their output conductance, the quality factor can be approximated by the following expression:

(7) $$Q=\frac{R_{p}}{\omega L_{\mathrm{eq}}}$$

Regardless of whether the circuit inherently exhibits a high R_p_ or achieves it through optimization techniques, the series resistance R_s_ remains the primary limiting factor affecting the quality factor. Therefore, the quality factor can also be estimated using the following expression:

(8) $$Q=\frac{\omega L_{\mathrm{eq}}}{R_{s}}$$

### 2.2. Proposed Grounded Tunable Active Inductor

**Figure 4:**
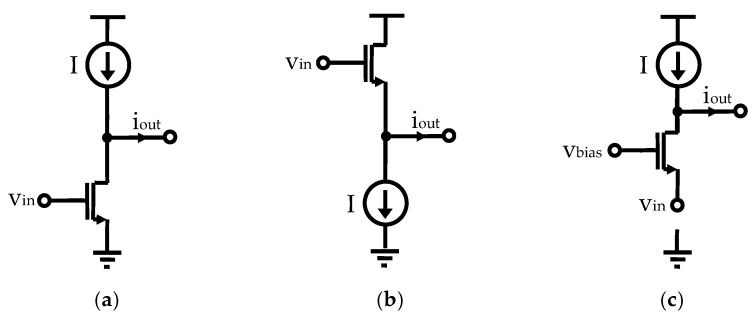
Fundamental transconductor configurations: (**a**) common source, (**b**) common drain, and (**c**) common gate.

The grounded active inductor topology [23,32] remains a widely adopted approach for emulating inductive behavior in integrated circuits, leveraging the well-established gyrator-C architecture. In this configuration, two active devices are arranged to generate an inductive input impedance, with the overall performance primarily determined by the choice of transconductor types. As shown in Figure 4, the fundamental transconductor configurations include the common-source (CS) structure exhibiting negative transconductance, and the common-drain (CD) and common-gate (CG) structures, which provide positive transconductance.

To address the limitations of conventional active inductor topologies, namely, low inductance values and a narrow frequency range for achieving high quality factors Q, this work employs an enhanced design strategy that integrates cascode and feedback-based techniques. Cascode and regulated cascode configurations are introduced to reduce the output conductance of the transistors, thereby decreasing the equivalent series resistance R_s_ and improving the quality factor [33]. To further enhance performance, a feedback resistor R_f_ is incorporated between the transconductance stages. This feedback path concurrently increases the equivalent inductance L_eq_ and reduces R_s_, thus enhancing the overall Q of the circuit [34]. As R_f_ also affects the self-resonant frequency and the frequency at which the maximum quality factor is achieved, i.e., *f*(Q_max_), making R_f_ tunable provides independent control of L, Q, and *f*(Q_max_), a key requirement for realizing a fully tunable active inductor (TAI).

**Figure 5:**
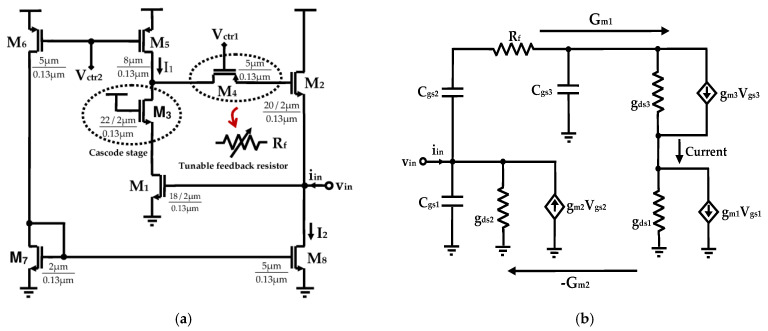
(**a**) Proposed grounded gyrator-C-based active inductor topology and (**b**) the corresponding small signal equivalent circuit.

The active inductor proposed in this work, along with its corresponding small-signal equivalent circuit, is depicted in Figure 5a,b. It consists of a single-port grounded structure, formed by two transconductors connected in a back-to-back configuration. Transistor M_1_ functions as a transconductor with negative transconductance in a common-source topology, while transistor M_2_ provides positive transconductance in a common-drain topology. All transistors operate in the saturation region to ensure proper biasing and linear operation.

A cascode transistor M_3_ is employed in the feedback path to enhance the quality factor Q by increasing the DC gain, resulting in a reduction in the series resistance R_s_. This reduction leads to a lower zero frequency, thereby broadening the effective inductive frequency range. In addition, transistor M_4_, operating in the linear region, functions as a feedback resistor R_f_. By decreasing R_s_ via the feedback resistance, the quality factor is further improved. The resistance can be tuned over a desired range by adjusting the gate voltage V_ctr1_.

The circuit shown in Figure 5a requires two current sources to bias transistors M_1_, M_3_, and M_2_. Transistors M_5_, M_6_, M_7_, and M_8_ serve as the source supply terminals, where M_5_ sources current I_1_, and M_8_ sinks current and supplies I_2_ to the active inductor.

The parallel capacitance C_p_, parallel resistance R_p_, series resistance R_s_, and equivalent inductance L_eq_ of the active inductor are derived as shown in Equations (9), (10), (11), and (12), respectively. For simplification, certain parasitic elements such as the impedance output of transistors and the gate–drain capacitance C_gd_ are neglected.

(9) $$C_{p}=C_{\mathrm{gs}1}$$

(10) $$R_{p}=\frac{1+g_{\mathrm{ds}3}R_{f}}{g_{\mathrm{ds}3}\left(2+g_{\mathrm{ds}3}R_{f}\right)}$$

(11) $$R_{s}=\frac{g_{\mathrm{ds}1}g_{\mathrm{ds}3}g_{m2}+\omega^{2}\left(C_{\mathrm{gs}2}^{2}g_{m3}-C_{\mathrm{gs}2}C_{\mathrm{gs}3}g_{m2}\left(1+g_{\mathrm{ds}3}R_{f}\right)\right)}{g_{m1}g_{m3}g_{m2}^{2}+\omega^{2}C_{\mathrm{gs}2}^{2}g_{m1}g_{m3}}$$

(12) $$L_{\mathrm{eq}}=\frac{C_{\mathrm{gs}2}g_{m2}g_{m3}+\omega^{2}C_{\mathrm{gs}2}^{2}C_{\mathrm{gs}3}\left(1+g_{\mathrm{ds}3}R_{f}\right)}{g_{m1}g_{m3}g_{m2}^{2}+\omega^{2}C_{\mathrm{gs}2}^{2}g_{m1}g_{m3}}$$

Assuming $g_{m2}^{2}>>\omega^{2}C_{\mathrm{gs}2}^{2}$, the equivalent inductance L_eq_ can be approximated by:

(13) $$L_{\mathrm{eq}}=\frac{C_{\mathrm{gs}2}g_{m2}g_{m3}+\omega^{2}C_{\mathrm{gs}2}^{2}C_{\mathrm{gs}3}\left(1+g_{\mathrm{ds}3}R_{f}\right)}{g_{m1}g_{m3}g_{m2}^{2}}$$

where g_ds1_, g_ds2_, and g_ds3_ denote the output conductances; C_gs1_, C_gs2_, and C_gs3_ represent the gate-source capacitances at nodes 1, 2, and 3, respectively; and g_m1_, g_m2_, and g_m3_ are the transconductances of transistors M_1_, M_2_, and M_3_, respectively.

From Equation (13), it is evident that the equivalent inductance exhibits an inverse relationship with the transconductance g_m3_, which is determined by the current flowing through M_3_. In this design, the cascode stage, implemented by transistor M_3_, is used to control the inductance. Lowering the gate voltage of M_3_ increases its drain current, which also impacts the drain current sourced by M_5_. Given that transconductance is defined as $g_{m}=\partial I_{D}/\partial V_{GS}=\sqrt{2\mu_{n}C_{ox}\frac{W}{L}I_{D}}$, an increase in drain current results in a higher g_m3_, thereby leading to a reduction in L_eq_.

The quality factor of the active inductor is given by:

(14) $$Q=\frac{C_{\mathrm{gs}2}g_{m2}g_{m3}\omega +\omega^{3}C_{\mathrm{gs}2}^{2}C_{\mathrm{gs}3}(R_{f}g_{\mathrm{ds}3}+1)}{g_{\mathrm{ds}1}g_{\mathrm{ds}3}g_{m2}+\omega^{2}(C_{\mathrm{gs}2}^{2}g_{m3}-C_{\mathrm{gs}2}C_{\mathrm{gs}3}g_{m2}(R_{f}g_{\mathrm{ds}3}+1))}$$

The zero frequency ω_z_ and the self-resonant frequency ω_0_, which establish the operational frequency boundaries, are expressed as:

(15) $$\omega_{z}=\frac{g_{\mathrm{ds}1}g_{\mathrm{ds}3}g_{m2}+\omega^{2}\left(C_{\mathrm{gs}2}^{2}g_{m3}-C_{\mathrm{gs}2}C_{\mathrm{gs}3}g_{m2}\left(1+g_{\mathrm{ds}3}R_{f}\right)\right)}{C_{\mathrm{gs}2}g_{m2}g_{m3}+\omega^{2}\left(C_{\mathrm{gs}2}^{2}C_{\mathrm{gs}3}\left(1+g_{\mathrm{ds}3}R_{f}\right)\right)}$$

(16) $$\omega_{0}=\sqrt{\frac{g_{m1}g_{m3}g_{m2}^{2}+\omega^{2}C_{\mathrm{gs}2}^{2}g_{m1}g_{m3}}{C_{\mathrm{gs}1}C_{\mathrm{gs}2}g_{m2}g_{m3}+\omega^{2}\left(C_{\mathrm{gs}2}^{2}C_{\mathrm{gs}1}C_{\mathrm{gs}3}\left(1+g_{\mathrm{ds}3}R_{f}\right)\right)}}$$

Since the additional inductive reactance introduced by the feedback resistor R_f_ appears in the expressions for both the quality factor Q and the equivalent inductance L_eq_, its influence varies as a function of this resistance.

**Figure 6:**
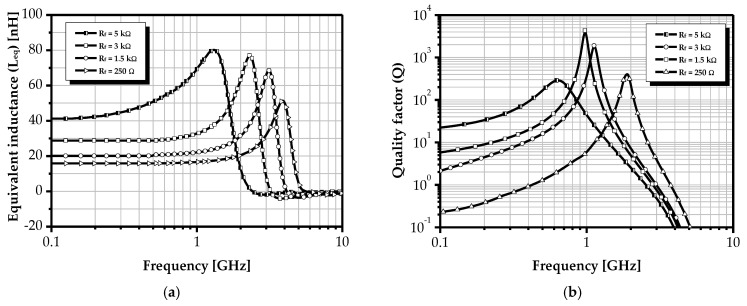
Effect of feedback resistor R_f_ on (**a**) equivalent inductance and (**b**) the quality factor.

As indicated by Equation (14), and from the associated Equations (11) and (12), the inclusion of a cascode stage contributes to a reduction in the equivalent series resistance, as g_m3_ < 1, thereby improving the quality factor. Moreover, the incorporation of a feedback resistor R_f_ significantly enhances Q by simultaneously increasing L_eq_ and reducing R_s_. By tuning R_f_, it is possible to adjust both the inductance and the quality factor. However, as shown by Equation (17) and illustrated in Figure 6, a substantial increase in R_f_ results in a pronounced reduction in *f*_Q_, the frequency at which the quality factor reaches its maximum. To mitigate this limitation, a secondary tuning mechanism is introduced via variation in the output conductance g_ds5_, which is controlled using the voltage V_ctr2_. Consequently, simultaneous tuning of V_ctr1_ and V_ctr2_ enables the realization of the desired inductance and high-Q performance at the target operating frequency *f*_Q_.

(17) $$f_{Q}\approx \frac{1}{2\pi }\sqrt{\frac{g_{m3}+g_{\mathrm{ds}1}g_{\mathrm{ds}3}}{C_{\mathrm{gs}2}\left[C_{\mathrm{gs}2}(R_{f}g_{\mathrm{ds}3}+1)+C_{\mathrm{gs}3}(2R_{f}g_{\mathrm{ds}3}+1)\right]}}$$

It is evident that both L_eq_ and Q are strongly dependent on the value of R_f_. Specifically, the inductance increases proportionally with R_f_. Simulation results indicate that the inductance varies from 51 nH to 80 nH over a frequency range of 1.32 GHz to 3.88 GHz. From the analysis of the corresponding plots, it is observed that the variation in the quality factor with frequency exhibits a parabolic profile within the inductive operating region. The quality factor increases with R_f_ until it reaches a maximum value of 3942 at 0.987 GHz. Beyond this point, despite continued increases in R_f_, the quality factor gradually decreases. This behavior can be explained by the increasing influence of resistive effects, particularly series parasitic resistance, which are minimal near the frequency where the quality factor is maximized but become more prominent outside that range. Additionally, increasing R_f_ causes a downward shift in the resonance frequency. These findings validate the effectiveness of the proposed method, maintaining a quality factor greater than 250 across the frequency range 0.62 GHz to 1.97 GHz.

The relationships between the principal circuit parameters considered in the parametric analysis and optimization process are summarized in Table 1 [35].

## 3. Experimental Results

**Figure 7:**
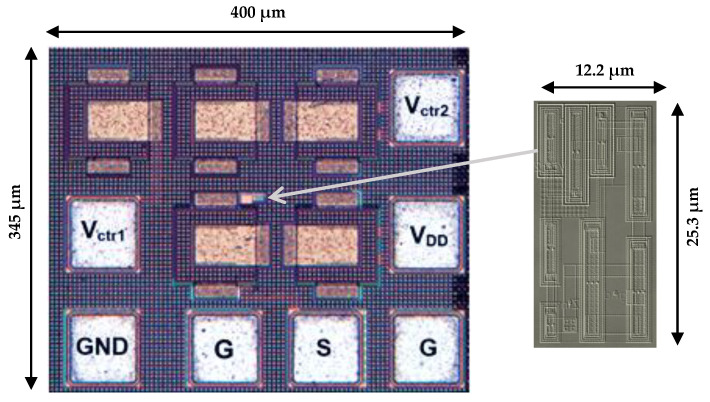
Die micrograph of the fabricated active inductor.

The proposed grounded tunable active inductor was implemented using 0.13 μm STMicroelectronics CMOS technology as proof of concept. Fabrication and characterization were conducted under nominal process–voltage–temperature (PVT) conditions. All presented results correspond to this typical corner. Measurements were carried out on 25 fabricated chips, which exhibited a tightly clustered performance distribution, thereby confirming the design’s consistency and reliability under standard operating conditions. Figure 7 shows the die photographs of a fabricated chip, occupying an area of 400 μm × 345 μm including bond pads and of 25.3 μm × 12.2 μm without bond pads.

**Figure 8:**
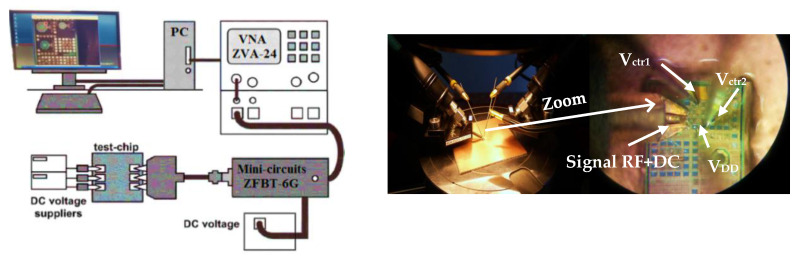
Experimental test-bench setup of the proposed active inductor.

The top right corner of Figure 8 depicts the active inductor during testing, while the left side illustrates the test-bench environment. To ensure accurate measurement results, performance metrics were analyzed after de-embedding the loading effects introduced by the input RF pads. The 20 pF decoupling capacitors were implemented on-chip. On-wafer measurements were performed using a Rohde & Schwarz ZVA-24 vector network analyzer (VNA) with RF and DC probing. The measurements were conducted under a 1 V supply with an input signal amplitude of –20 dBm (27°) at 2 GHz. The measured power consumption of the grounded active inductor was 2.0 mW, primarily attributed to the gyrator-C core.

**Figure 9:**
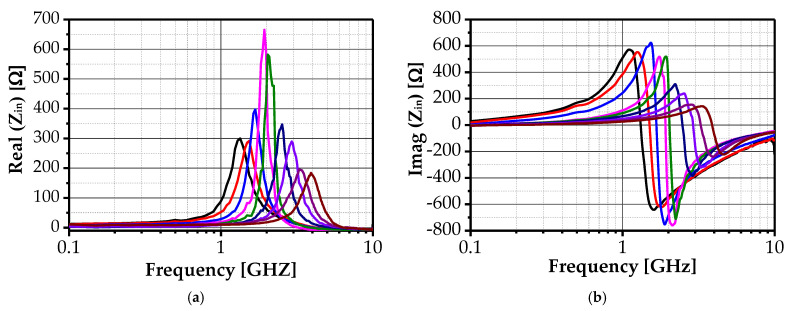
Measured input impedance of the proposed AI: (**a**) real part and (**b**) imaginary part.

One-port S-parameter measurements obtained from the VNA demonstrate the variation in the magnitude and self-resonant frequency of Z_11_ as functions of the control voltages V_ctr1_ and V_ctr2_. As depicted in Figure 9a,b, sweeping V_ctr1_ from 0.3 V to 0.54 V and V_ctr2_ from 0.39 V to 0.47 V enables the self-resonant frequency tuning of the *Z*_11_ imaginary part from 1.08 GHz to 3.9 GHz and of the *Z*_11_ real part from 1.337 GHz to 3.96 GHz. This tunability is accompanied by variations in the magnitude of the impedance components, validating the inductor operational flexibility and the adopted reconfiguration methodology effectiveness.

**Figure 10:**
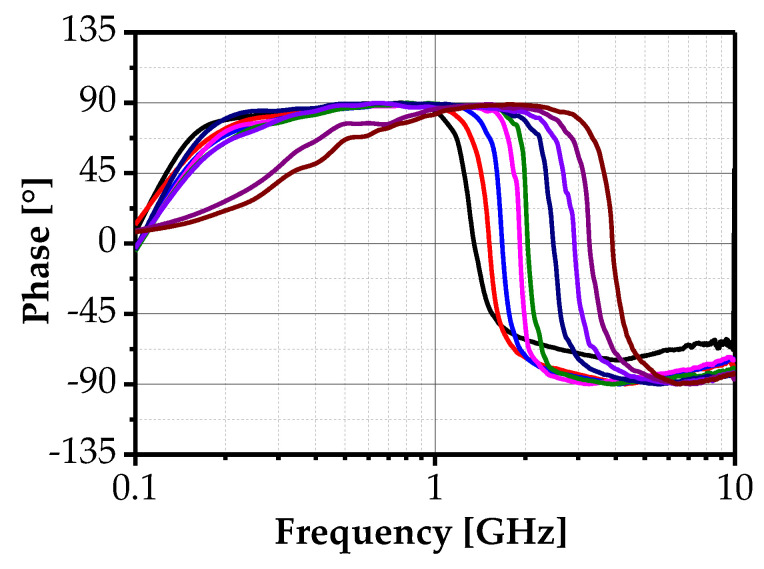
Measured phase response of the input impedance for the proposed AI.

Figure 10 presents the measured phase of the input impedance, delineating the operational frequency range of the inductor. The circuit begins exhibiting inductive characteristics at 87 MHz (ω_z_), with the phase angle reaching 45° at 150 MHz and peaking near 89°, maintaining inductive behavior up to 3.96 GHz (ω_0_), where the phase returns to 0°.

**Figure 11:**
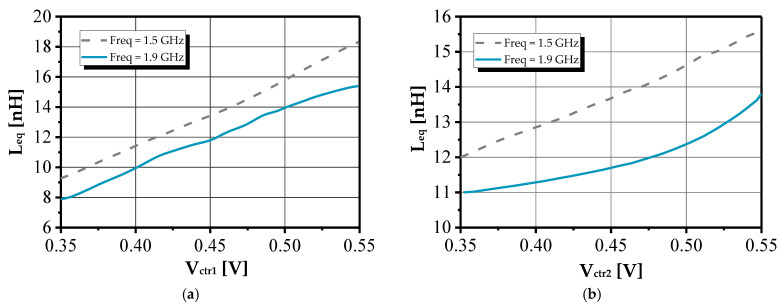
Variation in equivalent inductance and quality factor as a function of (**a**) V_ctr1_ with constant g_ds5_ and (**b**) V_ctr2_ with fixed R_f_.

Based on the measured S-parameters, both the equivalent inductance and the quality factor Q were extracted, providing further validation of the inductor’s performance. Figure 11 illustrates the influence of control voltages V_ctr1_ and V_ctr2_ on the equivalent inductance, with V_DD_ = 1 V. Measurements were conducted under two conditions: with R_f_ fixed and with the conductance g_ds5_ held constant. The equivalent inductance L_eq_ exhibits linear tuning behavior, as shown in Figure 11a,b, with the quality factor peaking at approximately 1.5 GHz and 1.9 GHz.

**Figure 12:**
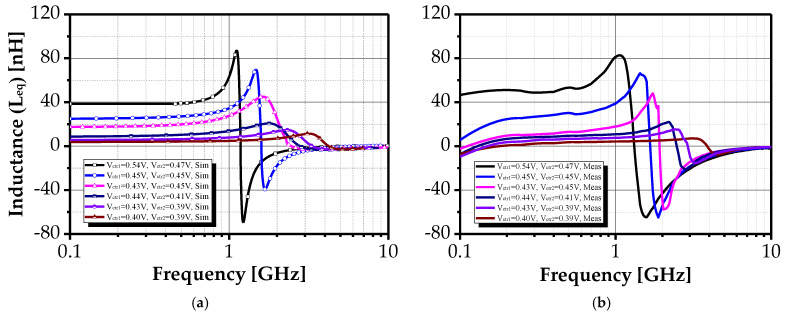
Input inductance values for the proposed AI’s input impedance under different control voltages V_ctr1_ and V_ctr2_: (**a**) simulated and (**b**) measured.

**Figure 13:**
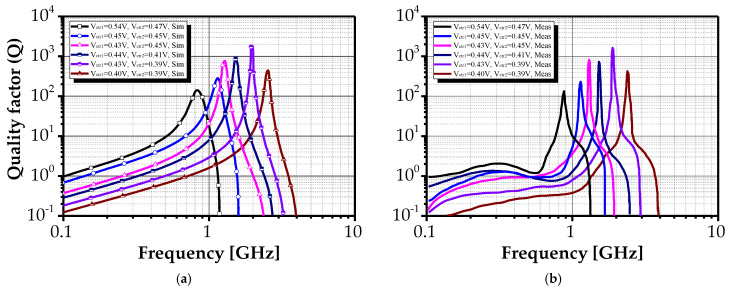
Quality factor for the proposed AI’s input impedance under various control voltages V_ctr1_ and V_ctr2_: (**a**) simulated and (**b**) measured.

Figure 12 further demonstrates that inductance values can be adjusted over a wide frequency range (668 MHz to 3.96 GHz), with corresponding inductance variation from 84.4 nH to 6.7 nH. Figure 13 presents measured quality factor values for various control voltage settings, showing high Q values (up to 50) across frequency-independent bands. The maximum recorded quality factor of 1586 was achieved at 1.9 GHz under bias voltages V_ctr1_ = 0.43 V and V_ctr2_ = 0.41 V. The measurement trends in Figure 11 and Figure 12 align with those discussed in §2.2, confirming the design’s expected behavior.

Nevertheless, due to parasitic effects in the fabricated structure, the measured values of L_eq_ and Q differ slightly from the simulation results. Such discrepancies are typical in practical implementations, where parasitic capacitances, resistances, and layout-related effects influence circuit behavior. The quality factor naturally varies with frequency due to the frequency-dependent characteristics of the transconductors. Slight shifts in equivalent inductance and self-resonant frequency were also observed, with some measured values exceeding simulation predictions.

A primary factor contributing to variations in the quality factor is the frequency-dependent behavior of the transconductors, wherein the transconductance g_m_ decreases slightly at higher frequencies, thereby affecting the impedance transformation. Additional degradation arises from parasitic capacitances, specifically the gate-source (C_gs_) and gate-drain (C_gd_) capacitances, along with the finite output resistance of the transistors, all of which introduce losses that reduce the quality factor across most frequencies. Furthermore, deviations in fabrication processes, such as reduced oxide thickness or variations in interconnect geometry, can alter effective parasitic capacitances, exacerbating this anomaly. In practical RFIC implementations, such fluctuations in the quality factor can impair circuit performance, leading to diminished selectivity in bandpass filters, increased insertion loss in impedance matching networks, and degraded phase noise characteristics in oscillators.

Table 2 provides a comparative performance analysis between the proposed active inductor fabricated in this work and previously reported designs in the literature. The proposed design achieves an inductance tuning range of 6.7 nH to 84.4 nH, significantly broader than those reported in prior works, which typically remain below 25 nH. Although the design in Ref. [28] reports an inductance of 191 nH, this value lacks experimental verification and thus remains to be validated. Furthermore, the proposed design achieves a maximum quality factor Q_max_ of 1586, which substantially exceeds other reported values, such as the value of 28 in Ref. [36] and the value of 45 in Ref. [37]. The power consumption of the active inductors in Refs. [37,38] is reported to be 21 mW and 16 mW, respectively, exceeding the 2 mW consumption of the proposed design by more than ten and eight times, respectively. A further advantage of the proposed inductor is its compact layout area of 308 µm^2^, which is significantly smaller than those of existing designs (typically ranging from 3200 to 8200 µm^2^), enhancing its suitability for integration in compact RF systems.

Nevertheless, certain trade-offs are inherent. The wide tunability and high Q performance render the design more sensitive to variations in bias voltage, necessitating accurate bias stabilization to ensure consistent operation under process and temperature fluctuations. This requirement may increase overall system-level design complexity. Furthermore, the design may exhibit reduced linearity at higher signal amplitudes, which could constrain its applicability in circuits demanding high linearity, such as precision low-noise amplifiers.

## 4. Conclusions and Future Works

In this paper, a high-quality CMOS grounded active inductor has been presented. It was optimized in 130 nm CMOS technology. By incorporating a feedback resistor into a cascode grounded configuration, the proposed design achieves significant improvements in both inductance and quality factor. Experimental validation has demonstrated a maximum quality factor of 1586 and an inductance tuning range of 6.7 nH to 84.4 nH, with a low power consumption of only 2 mW.

The inductive bandwidth of the design is tunable across a frequency range of 87 MHz to 3.96 GHz, offering high versatility for diverse receiver applications. It is compatible with multiple mobile communication bands, including 4G and 5G NR (FR1) bands (n1, n3, n5, n7, n8, n20, n28, n38, n41, and n78), as well as the 2.4–2.5 GHz ISM band, enabling its integration into wireless system designs.

Our future work will be focused on further optimizing the design to extend its frequency tuning range and enhance its integration with advanced circuit architectures, aiming to improve overall performance and adaptability.

## Figures and Tables

**Table 1:** Optimization summary of tunable active inductor performance based on transconductance and feedback resistance variation.

**g_m_**	**ω_z_**	**ω_0_**	**R_s_**	**L_eq_**	**Q**
**+**	↓	↑	↓↓	↓↓	↑↑
**−**	↑	↓	↑↑	↑↑	↓↓
**R_f_**	**ω_z_**	**ω_0_**	**R_s_**	**L_eq_**	**Q**
**+**	↓↓	↑	↓	↓	↑↓
**−**	↑↑	↓	↑	↑	↓↑

**Table 2:** Performance comparison summary of state-of-the-art active inductor implementations.

Ref.	Topology	Verification	L_eq_ (nH)	Q_max_	SRF (GHz)	P_DC_ (mW)	Area (µm²)	CMOS Process (nm)	V_DD_ (V)
[25]	Gyrator-C	Meas.	5.7	70	2.5	8	88 × 90 ^+^	180	2.0
[28]	Gyrator-C	Sim.	191.7	286	4.81	-	-	65	1.2
[36]	Gyrator-C	Meas.	27	28	1.5	4	0.1 × 0.1 mm^2^ *	180	1.8
[37]	Gyrator-C	Meas.	0.65–1	45	>8	21	390 × 290 ^+^	130	1.5
[38]	Gyrator-C	Meas.	0.8–3.5	70	3.5	16	8800 ^+^	180	3.3
[39]	Gyrator-C	Meas.	2.5–5	740	>6	-	-	180	1.6
[40]	Gyrator-C	Sim.	3.55–26	895	5.5	0.5	22 × 27.5 ^+^	90	1.0
[41]	Gyrator-C	Meas.	22	450	10	3.6	0.2 × 0.3 ^+^	65	1.2
This work	Gyrator-C	Meas.	6.7–84.4	1586	3.96	2	12.2 × 25.3 ^+^	130	1.0

^+^ Without bond pads. * With bond pads.

## Data Availability

Data are contained within the article.

## Abbreviations

The following abbreviations are used in this manuscript:

RF	Radio frequency
CMOS	Complementary metal-oxide semiconductor
AI	Active inductor
GSG	Ground–signal–ground
SRF	Self-resonant frequency
NR	New Radio
FR1	Frequency Range 1
5G	Fifth generation
FDD	Frequency Division Duplex
TDD	Time Division Duplex
LNA	Low-noise amplifier
VCO	Voltage-controlled oscillator
NIC	Negative impedance converter
FinFET	Fin field-effect transistor
SOI	Silicon-on-insulator
GaN	Gallium nitride
KCL	Kirchhoff’s Current Law
CD	Common drain
CG	Common gate
CS	Common source
TAI	Tunable active inductor
DC	Direct current
VNA	Vector network analyzer
PVT	Process–voltage–temperature
4G	Fourth generation
ISM	Industrial, scientific, and medical
